# On the plausibility of socioeconomic mortality estimates derived from linked data: a demographic approach

**DOI:** 10.1186/s12963-017-0143-3

**Published:** 2017-07-14

**Authors:** Mathias Lerch, Adrian Spoerri, Domantas Jasilionis, Francisco Viciana Fernandèz

**Affiliations:** 1 Max Planck Institute for Demographic Research, Demographic Research Centre, Vytautas Magnus University, Rostock, Germany; 2 Institute of Social and Preventive Medicine, University of Bern, Bern, Switzerland; 3 Institute of Statistics and Cartography of Andalusia, Andalusia, Spain

**Keywords:** Mortality, Socioeconomic differentials of mortality, Linked data, Quality assessment, Indirect estimation, Andalusia, Finland, Lithuania, Switzerland.

## Abstract

**Background:**

Reliable estimates of mortality according to socioeconomic status play a crucial role in informing the policy debate about social inequality, social cohesion, and exclusion as well as about the reform of pension systems. Linked mortality data have become a gold standard for monitoring socioeconomic differentials in survival. Several approaches have been proposed to assess the quality of the linkage, in order to avoid the misclassification of deaths according to socioeconomic status. However, the plausibility of mortality estimates has never been scrutinized from a demographic perspective, and the potential problems with the quality of the data on the at-risk populations have been overlooked.

**Methods:**

Using indirect demographic estimation (i.e., the synthetic extinct generation method), we analyze the plausibility of old-age mortality estimates according to educational attainment in four European data contexts with different quality issues: deterministic and probabilistic linkage of deaths, as well as differences in the methodology of the collection of educational data. We evaluate whether the at-risk population according to educational attainment is misclassified and/or misestimated, correct these biases, and estimate the education-specific linkage rates of deaths.

**Results:**

The results confirm a good linkage of death records within different educational strata, even when probabilistic matching is used. The main biases in mortality estimates concern the classification and estimation of the person-years of exposure according to educational attainment. Changes in the census questions about educational attainment led to inconsistent information over time, which misclassified the at-risk population. Sample censuses also misestimated the at-risk populations according to educational attainment.

**Conclusion:**

The synthetic extinct generation method can be recommended for quality assessments of linked data because it is capable not only of quantifying linkage precision, but also of tracking problems in the population data. Rather than focusing only on the quality of the linkage, more attention should be directed towards the quality of the self-reported socioeconomic status at censuses, as well as towards the accurate estimation of the at-risk populations.

## Background

Knowledge of socioeconomic differentials in mortality is crucial for monitoring public health. It also informs the policy debate about social inequality, social cohesion and exclusion, as well as about the equitable flexibilization of retirement ages in the context of the reforms of pension systems. Because information on socioeconomic status on death certificates is very problematic or absent, the most proper way is to identify this status from information reported by individuals at a prior census, or to derive it from specific registers (e.g., educational registers). This is usually achieved by means of the linkages between death and population records, as implemented in more than a dozen OECD countries since the 1950s [1, 2].

The linkage is straightforward when observations are indexed in both databases by a common identifier variable (such as a social security number), enabling a deterministic linkage. Otherwise, the identification of observations referring to the same individual requires comparison of each pair of records (death and population) according to a common set of discriminating information (such as sex, date and place of birth, etc.). The pair of records with the most likely correspondence is linked if they reach a minimum threshold of agreement across the linkage variables (i.e., probabilistic linkage) [3]. Our objective is to assess from a demographic perspective the plausibility of the estimated mortality levels according to socioeconomic status derived from linked data.

Previous assessments of linked data have used three main approaches. The first approach is only applicable when information on socioeconomic status is available on both the death certificates and the population data: the aim is to assess the quality of the information on the death certificate by comparison with that retrieved from the census using record linkage [4–8].

The second and third approaches aim at assessing the quality of the linkage procedure, which depends on the trade-off between two goals: linking the maximum number of deaths to population records (i.e., linkage sensitivity) in order to limit underestimation of mortality, and linking the deaths to the right living individuals (i.e., linkage precision) in order to correctly estimate mortality levels by socioeconomic status [9]. Although poor linkage sensitivity underestimates mortality, the mortality gradients are only affected when the sensitivity differs by socioeconomic status. The problem can also be resolved by linking (or imputing) the unlinked deaths from a first linkage round in a subsequent round using more relaxed criteria. Poor linkage precision, by contrast, leads to a misclassification of deaths according to socioeconomic status. This produces a more important bias because it misestimates mortality of different population strata in different directions, so that the gradients are always biased.

These problems with the quality of the linkage have been tackled, on the one hand, by analysis of the sociodemographic profile of the unlinked when compared to the linked deaths, and on the other hand, by analysis of several sets of the same deaths which have been linked according to different linkage thresholds [10–12]. These two approaches provide information on how the probabilistic method affects the socioeconomic mortality gradient. However, they do not provide quantitative measures of linkage sensitivity and precision by socioeconomic status. This is only possible if the true linkage status is known, e.g., if a subset of records can be matched using a unique identifier [11, 13]. What can be done if neither such a gold-standard (knowing the true link status), nor external mortality estimates based on unlinked data, are available for comparison, as is the case in several countries?

Another limitation of previous approaches is that they only validate the numerator of mortality rates by socioeconomic status. The accuracy of the denominators has never been assessed. In statistically developed countries, misestimation of at-risk populations may arise when the socioeconomic data are collected only for a sample of the population (to which the deaths can be linked). In this case, the data need to be adjusted using sample weights that are usually calculated based on the total reference population (by age and sex) and may not provide representative figures for subregions or subpopulations. The at-risk population may also be misclassified according to socioeconomic status due to changes in the methodology of data collection that lead individuals to provide wrong socioeconomic information (e.g., changes in the mode of interaction between interviewer and respondent, and in the formulation of questions and response items, etc.).

To assess the plausibility of socioeconomic mortality levels derived from linked data, we propose a procedure based on indirect demographic estimation, which enables us not only to quantify the quality of the linkage by socioeconomic status, but also to track problems with the population data. We use death distribution methods to evaluate whether the numbers of deaths linked to different socioeconomic strata of the population are consistent with the changes in the population counts over time [14, 15]. The objective is to investigate whether the mortality estimates are affected by the misestimation and/or misclassification of the at-risk population according to socioeconomic status, on the one hand, and/or by the misclassification of deaths that results from their over- and under-linkage to different population strata, on the other hand. For the application of the method we chose three countries and one autonomous region in Europe, which cover various quality issues, ranging from deterministically to probabilistically linked deaths with different problems in the population data. The next section introduces the data, the method and our assessment strategy. We then present the results and discuss the data contexts in which death distribution methods provide a relevant quality assessment. Our results also have implications for the production and the use of linked mortality data.

## Methods

### The linked mortality data

We used linked mortality data for the population aged 60 and above from Finland, Lithuania, Switzerland, and the autonomous region of Andalusia (Spain), covering the period from 1980 to 2011 (see Table 1). The registration of deaths and (enumeration of) population is virtually complete in all of these settings.

**Table 1:** Linked mortality data used for the assessment of the plausibility of old-age mortality estimates by educational attainment (60+), Europe, 1980–2011

Country/region	Period covered by deaths at age 60 and over	Population aged 60 and over at start of interval	% low-skilled	% high-skilled	Source for population at risk	Source of the information on education, linked to the deaths	Death linkage method	Linkage rate
Finland	1980–2000	781,602	85.0	4.3	Annual population register, linked to register of aliens	Annual education register (linked to population register)	Deterministic (ID)	100%
Lithuania	2001–2011	672,186	66.5	9.4	Census 2001 & 2011	Census 2001	Deterministic (ID)	94%
Switzerland	1991–2000	1,310,978	48.5	15.0	Census 1990 & 2000 (& linkage of intercensal survivors)	Census 1990 (& Census 2000 for survivors)	Probabilistic and deterministic (for most discriminant records)	94%
Andalusia (Spain)	2002–2011	1,364,081	82.3	3.9	Census 2001 & weighted sample Census 2011; updated population register, linked to migration register	Census 2001	Deterministic (ID)	98%

We assess the accuracy of old-age mortality rates by socioeconomic status using educational attainment as a proxy because it should not vary within individuals over time, and is available for most persons (including for women and older people).

We rely on the aggregated data from Statistics Finland, which used a personal identification number (ID) to deterministically link 99% of the deaths over the period 1980 to 1999 with records from the population register. The population register is updated annually through deterministic linkages with earlier records and with the migration register. Information on educational attainment is obtained and annually updated through deterministic linkage with the Register of Completed Education and Degrees (using the same ID). This Register is based on the data on educational qualifications and degrees as of the 1970 Census, which was further updated and corrected annually on the basis of information directly obtained from educational institutes (including qualifications and degrees attained abroad). The education-specific person-years exposed to the risk of mortality can thus be accurately estimated on an annual basis.

Statistics Lithuania deterministically linked 94% of the deaths having occurred between April 2001 and February 2011 to records from the census in February 2001, using a personal ID. The remaining 6% of unlinked deaths have been excluded from this assessment. The linkages were performed by employees of Statistics Lithuania who have permits to work with individual data. The data used in this study were provided in an aggregated multidimensional frequency table format that combines deaths and population exposures and are split by sociodemographic variables, including age, sex, education, marital status, ethnicity, and urban-rural residence. The at-risk populations by educational attainment during the 2000s have been estimated based on the average counts at the 2001 Census and the subsequent census in March 2011.

We also rely on the Swiss National Cohort database (SNC) [16, 17]. In order to first identify intercensal survivors and migrants, individual records from the census in December 1990 were linked deterministically and probabilistically to the census in December 2000 and to the Central Register of Foreigners, respectively. In a second step, 94.3% of the deaths from the mortality registry 1991–2000 were probabilistically linked to those records of the 1990 Census that were not identified as belonging to a survivor or emigrant. The remaining unlinked deaths at this stage were then linked using a more pragmatic procedure [18], and are also included in this assessment. The education-specific at-risk populations can be estimated based on the average of the counts at the 1990 and the 2000 Censuses. As intercensal survivors have been identified through record linkage across the two censuses, we are able to assess the consistency over time of the self-declared information about educational attainment. The linkage team also applied harmonization rules for each variable, to ensure a consistent use of the data.^1^

For Andalucia, we use the Base de Datos Longitudinal de Población [19]. Based on an ID, the Institute of Statistics and Cartography of Andalusia deterministically linked 98% of the deaths having occurred over the period 2002–2011 to the population register *Padron*. The *Padron* was previously updated by matching records from one year to the next, as well as to the registers of demographic events. For 99% of these populationS (and linked deathS) records, information on education was then obtained by deterministic linkage with the records from the 2001 Census. The data used in this study were provided by the Institute of Statistics and Cartography of Andalusia in an aggregated multidimensional frequency table format that combines deaths and population exposures, and are split by sociodemographic variables. The education-specific at-risk populations of Andalucia can be estimated according to two methods. The first is based on the average population counts at the 2001 Census and the weighted counts at the 2011 10%-sample Census. Weighting factors are provided to get representative statistics for the whole of Spain. The second method relies on the annual population register (linked to the 2001 Census). Furthermore, Institute of Statistics and Cartography of Andalusia linked the records across the two censuses for us in order to investigate changes over time in the responses to the question about educational attainment. However, this information cannot be harmonized for the entire population, because the 2011 Census covered only a sample of the population.

Based on this description of the four data sets, we expect to find a perfect linkage precision and estimation of the education-specific at-risk population in Finland, where neither the deterministic linkage nor the annually updated population and education registers should bias the mortality estimates. The census-based denominators of mortality rates in Lithuania, by contrast, may be affected by educational misclassification of the at-risk population over time. Similar biases can be expected for Switzerland. In this country, the probabilistic linkage of deaths may also lead to their misclassification according to educational attainment. In Andalucia, the census-based education-specific estimates of the at-risk populations may be biased not only because of changes in the educational classification of the enumerated population, but also because of problems with the weighting of the 2011 sample-Census counts.

### The synthetic extinct generation method

To validate mortality estimates by educational attainment as derived from linked data we rely on death distribution methods, which exploit the mathematical relationships between age-specific counts of living individuals at two dates and the deaths occurring during the interval [14, 15].

We use the Synthetic Extinct Generation (SEG) method. It has been designed to estimate the rate of completeness of death registration, relative to the completeness of census enumeration [20, 21]. The rationale of the SEG method is straightforward: the number of individuals born in a past year (i.e., a cohort) and reaching exact age *a* in a current period *t* (*N(a)*) is equal to the number of deaths that this cohort is experiencing at higher ages in later periods, until the last survivor dies (*N* ^*d*^ *(a)*) [22]. Comparison of these two numbers provides an estimate of the completeness of death registration – provided that migration is a negligible component of population change.

To apply this assessment to period deaths, the idea behind the SEG method is to approximate the number of future age-specific cohort deaths under the assumption of a stable population (i.e., only the number of births varies over time, with constant age-specific risks of mortality and no migration during the observation period) [14, 23]. In this context, future cohort deaths can be approximated by adjusting the age-specific number of period deaths for the demographic history of the population, as synthetized by the period age-specific growth rates (see also Methodological appendix):

$$ {N}^d(a)={\int}_0^w{D}^{\ast}\left( x+ y\right)\mathit{\exp}\left[{\int}_0^y r\left( x+ z\right) dz\right] dy $$

Where *a* stands for exact age; D*(x) is the observed number of period deaths and *r(x)* the annual growth rate at age *x* in the period *t*. Growth rates can be estimated from two successive age-specific counts of the population – such as censuses at the beginning and the end of *t*, or population registers. Thus, the age-specific completeness of population counts is also assumed to be constant over time. Violation of this and the stable population assumptions can be detected in the results and, therefore, do not bias the assessment (see further down and [23]).

### Tracking the socioeconomic misclassification of deaths

In the context of complete death registration, the SEG method can be used to estimate the education-specific linkage rate of deaths to the total population. We applied the method separately to each educational subpopulation by sex. The death-based number of e.g., tertiary educated cohort survivors to exact age *a* in an intercensal period *t* (*N* ^*d*^_*TERT*_ *(a)*) is approximated by summing the following products for each age group *x* (above *a*):

(i.) the age-specific *deaths* in *t* having been *linked to tertiary educated individuals at the first census* (*or the population register*) and
(ii.) the exponentiated sum of age-specific *growth rates* in *t* of the tertiary educated population, as estimated *from successive age-distributions (at censuses or from the population register) using unlinked data*.

**Fig. 1:**
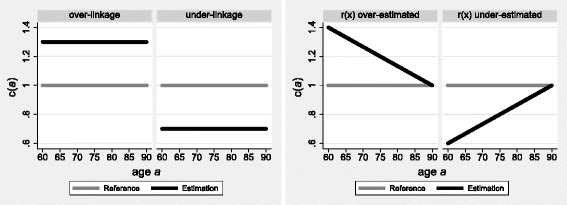
Stylized patterns of linkage rates of deaths for two strata of an exhaustive population

The fraction of deaths above age *a* of the tertiary educated population that could be linked to the respective population records (*c*_*TERT*_ *(a)*) is obtained by dividing this *death-based estimate* of cohort survivors (*N* ^*d*^_*TERT*_ *(a)*) by a *population-based estimate* (*N*_*TERT*_ *(a)*). The population-based estimate is approximated from successive age-distributions of the tertiary educated populations (at censuses or from the register) *using unlinked data*. All deaths of the tertiary stratum have been linked to the respective population records when the set of *c*_*TERT*_ *(a)* computed for successive ages *a* follows a straight horizontal line at unity. Over-linkage of deaths is indicated when the straight line falls above unity. Under-linkage of deaths is indicated when the straight line falls below unity (see upper panel of Fig. 1).

When all registered deaths have been linked (or imputed) to the population, these education-specific linkage rates provide an estimate of education-specific linkage precision: over-linkage of deaths to the tertiary educated population must coincide with an under-linkage of deaths to another educational stratum, and vice-versa (see upper panel of Fig. 1). In the presence of remaining unlinked deaths, the results confound the effects of linkage precision and sensitivity. Yet if age and sex are used as blocking variables in the linkages procedure (a common strategy), and the linkage sensitivity can be assumed not to differ by education, the precision of linkage may be assessed by comparison of the education-specific sets of *c(a)* against the reference estimates computed for the total population (rather than against the hypothetical line at unity). Consequently, the education-specific sets of *c(a)* provide information on the misclassification of the linked deaths according to educational attainment.

### Tracking the misclassification or misestimation of the at-risk population according to socioeconomic status

Problems in the socioeconomic data can be detected through the distinctive age patterns of *c(a)*, which arise from the violations of the SEG method’s simplifying assumptions. When the method is applied to all registered deaths and the total population, linearly increasing trends in *c(a)* over successive ages indicate that the age-specific growth rates (used to approximate future cohort deaths from current period deaths) are under-estimated. Linearly decreasing trends in *c(a)*, by contrast, reveal an over-estimation of growth rates (see Methodological appendix and [23]). This results either from different levels of completeness between the two censuses, or from the presence of significant migration flows during the observation period.

The interpretation of these distinctive age patterns in the results is different when the SEG method is applied to linked mortality data separately by educational attainment, and when migration is negligible (i.e., above a certain age) and census enumeration is virtually complete (as in most OECD countries). In this context, the linearly increasing or decreasing age-patterns in *c(a)* identify under- respectively over-estimation of different educational strata among the exhaustive total population at the second census, relative to the first one. Moreover, a linearly decreasing trend in *c(a)* for the high-skilled population (indicating its relative over-estimation at the second census) must coincide with a linearly increasing trend in *c(a)* for another educational stratum (which is under-estimated; see lower panel of Fig. 1). Thus, the method tackles the misestimation of the at-risk populations, which arises from problems in the educational classification over time or from problems with the demographic estimation per se.

We use the SEG method, instead of another death-distribution method (i.e., the General Growth Balance method), because it performs best in identifying problems with the completeness of population enumeration (or the classification/estimation of population according to socioeconomic status), and provides more robust estimates of death registration completeness [14, 15]. Further methodological details can be found in the Methodological appendix and the recent manuals edited by the United Nations Population Division [23] and Population Fund [24].

### Analytical strategy

During the periods of observation, the Swiss, Andalucian, and Lithuanian populations were characterized by important migration flows at young adult ages. The method is therefore applied to male and female deaths occurring at the ages 60 and above. We stratified the populations and deaths into three broad educational classes according to the national practices: tertiary level, secondary (post-compulsory) school level, and primary (compulsory) school level or lower. The missing information is also treated in accordance with national practices.^2^

Compared to the Lithuanian and Finnish data, the Swiss and Andalusian data enable a more detailed quality assessment. In Switzerland, we first assess the data using the original information on educational attainment (as stated at the two censuses). The second assessment uses the harmonized educational variable in order to evaluate the extent to which the misclassification of population affected the results. We then replicate the assessment for only the population born in the country and their linked deaths, in order to evaluate the sensitiveness of the results to potential emigration of foreigners around the retirement age, which would misestimate the education-specific at-risk populations.^3^

In Andalucia, a first set of assessments is based on the education-specific at-risk populations as estimated from the censuses. The effect of misestimation of populations due to immigration around retirement ages is then investigated by comparison with a replicated assessment based only on the non-migrant at-risk populations. These were estimated by taking into account in 2011 only the enumerated population who reported having already resided in the autonomous region in 2001. These census-based results are then compared with a final assessment, which is based on the at-risk populations as estimated from the register data, in order to document potential estimation biases related to the weighting of the 2011 sample-census counts.

## Results

### Finland

**Fig. 2:**
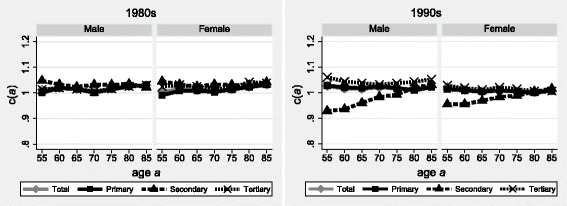
Linkage rates of deaths above age *a* according to sex and educational attainment, Finland, 1980–2000. Source: Register-linked mortality data Finland, 1980–2000. Note: educational attainment as based on educational register data

Results for Finland in the 1980s confirm an excellent precision of the linkage (Fig. 2): the age-specific sets of deaths-to-population linkage rates *c(a)* for all three educational strata align on a straight horizontal line slightly above unity. This is confirmed in the 1990s by the results for the lowest and highest educated population strata, especially among women. But the *c(a)* for the median educational strata are found to increase slightly but steadily from 94% among men (95% among women) at age 60, to linkage rates slightly above unity at age 85. This indicates a slight under-estimation of the population growth rates used to extrapolate future cohort deaths (*N* ^*d*^ *(x)*) from current period deaths. The population holding a secondary education diploma may be underestimated by the register due to the lack of complete accounting of immigration.

The overall results, however, are more than satisfactory, which was to be expected given the deterministic linkage of deaths and the availability of high quality register data to accurately estimate the education-specific person-years exposed to the risk of mortality. This application therefore supports the usefulness of the SEG method for our purpose.

### Lithuania

**Fig. 3:**
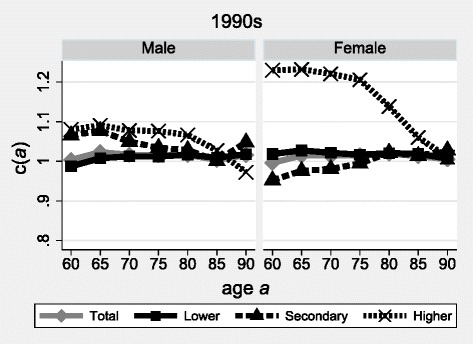
Linkage rates of deaths above age *a* according to sex and educational attainment, Lithuania, 2001–2011. Source: Census-linked mortality data Lithuania, 2001–2011. Note: educational attainment as based on censuses

In Lithuania in the 2000s, the sets of *c(a)* align on a straight line at unity for the total male and female populations, for the majority subpopulation holding the lowest educational level, and for the secondary education stratum (see Fig. 3). Overall, the quality of the linked data is satisfactory, which was to be expected given the deterministic linkage method.

However, the set of *c(a)* referring to the highest-educated populations form a straight line at 122% among women (109% among men) between ages 60 and 80 (75), and then linearly decline towards unity at age 90. This can be explained by the over-estimation of the intercensal growth rates of the population holding the highest educational level at ages 75–89 (up to an implausible 20% annually for women; not shown). The general census methodology remained the same in 2001 and 2011 (face-to-face interviews of the exhaustive population). But the question about educational attainment changed slightly, which may have led to inconsistent information over time.

Some people aged 75–89 probably selected the new tertiary school items in the 2011 questionnaire, whereas they indicated a secondary education level in 2001. This misclassification would have led to the overestimation of the tertiary stratum and to the underestimation of the secondary stratum in the 2011 Census, relative to 2001. The respective age-specific growth rates would have been biased accordingly. This interpretation of the data is supported, at least for women, by the inversed linear trends in *c(a)* for secondary and tertiary educated populations: the increasing estimates of *c(a)* for the secondary stratum (from below unity to levels slightly above unity) indeed mirrors the decreasing estimates among the tertiary educated. Given that the tertiary stratum represents a very small proportion of the total population at ages 75–89 (less than 5% among women and 8% among men; not shown), a small number of misclassified living individuals can have a large impact on the intercensal growth rates and, thus, on the estimates of *c(a)*.

### Switzerland

**Fig. 4:**
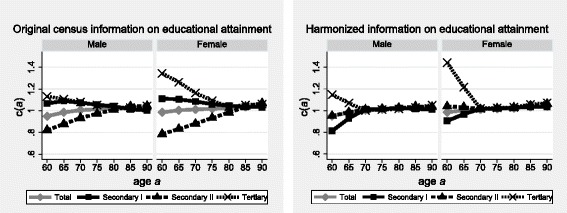
Linkage rates of deaths above age *a* according to sex and educational attainment, Switzerland, 1990–2000. Source: SNC 1990–2000. Note: educational attainment according to original and harmonized information from censuses

The upper panel of Fig. 4 shows the results for Switzerland in the 1990s, based on the original census information about educational attainment. The patterns are similar to those observed in Lithuania, although much more pronounced. Among men and women with secondary (post-compulsory; Sec II) education, the trend in *c(a)* increases linearly with age, from an apparent under-linkage (80%) at age 60 to an almost perfect linkage at age 90 (105%). The ratios *c(a)* for the tertiary and primary (compulsory; Sec I) educational strata, by contrast, start above unity and linearly decrease over the same age range (from 113% among men and 135% among women to 105%). These increasing and decreasing age patterns in the education-specific results indicate a misclassification of population according to educational attainment: the secondary stratum is under-estimated at the second census (relative to the first one), whereas the primary and tertiary strata are over-estimated.

The changes in the methodology of census enumeration were indeed important. In 1990, the paper-based questionnaire was sent home for self-administration by household members, and census operators collected the questionnaires door-to-door while verifying their completeness. In 2000, the questionnaires were either returned by mail to the Statistical Office or filled in online. Generally, the percentage of missing information increased [25]. Despite the efforts of the Swiss Federal Statistical Office (SFSO) to impute the missing information, the remaining percentage of records with missing educational attainment increases sharply with age among the enumerated populations in 2000: educational attainment was unknown for 7% of women and 9% of men aged 60–64 against more than 20% among both sexes at ages 90 and above (not shown).

Furthermore, the numbers and labels of response categories to the question regarding educational attainment changed between the censuses; from seven in 1990 to 10 different items in 2000. People indeed responded differently over time, as revealed by the comparison of the intercensal survivors’ stated information in 1990 and in 2000 (Table 2; moreover, the SFSO may have done false imputations). Among males, the percentage of inconsistent responses increases with lower educational levels mentioned in 1990. Only 65% of those holding a primary level confirmed this response in 2000, and for 22% information was missing (and not imputed by the SFSO). Answers were congruent for 77% of the tertiary educated, whereas 16% of them indicated a secondary level in 2000. Among female survivors, however, this “educational mobility” increased with the educational attainment reported in 1990. Indeed, of those who stated a primary level diploma in 1990, 20% and 9% provided no response at all and stated a secondary level, respectively, in 2000; 30% of the tertiary educated classified themselves as having a primary level and 10% mentioned a secondary level diploma in 2000. These patterns of “educational mobility” were slightly more pronounced among the population aged over 80 (not shown).

**Table 2:** Intercensal survivors’ stated educational attainment at the 2000 Census as a percentage of their self-classification at the 1990 Census, linked persons aged 60 and older by sex, Switzerland 1990 and 2000

Educational attainment in 1990	Educational attainment in 2000 (in %)					
	Primary	Secondary	Tertiary	No information	Total	Numbers
Men						
Secondary I	64	12	2	22	100	148,408
Secondary II	17	67	7	9	100	290,615
Tertiary	3	16	77	4	100	117,665
No information	42	20	6	33	100	9728
Women						
Secondary I	70	9	1	20	100	397,971
Secondary II	23	63	4	10	100	317,474
Tertiary	10	30	53	7	100	35,262
No information	53	13	2	32	100	23,207

Source: SNC 1990–2000

Thus, the estimates of the education-specific populations at risk to mortality are biased due to the discrepancies in individual reporting over time, the potentially false imputations of the missing information by the SFSO, and researchers’ rules applied to allocate the remaining missing cases to the known but misclassified population by education in 2000.

To evaluate the impact of the changes in the census methodology and questionnaire, the lower panel of Fig. 4 shows the results of the SEG-method applied to linked deaths and enumerated populations of Switzerland, which have been re-tabulated according to the harmonized educational variable. In contrast to the upper panel of Fig. 4, all ratios *c(a)* referring to ages 70 to 90 are now close to unity in the lower panel. In Switzerland, four fifth of all deaths occurring above age 60 are concentrated in this age range. The results therefore confirm a precise linkage of deaths of each educational stratum among the total population – despite the probabilistic method used.

Below age 70, the reclassification of the population according to the harmonized educational variable produced changing patterns of linkage precision. The lower panel of Fig. 4 indicates a shift from over- to under-linkage of deaths of the primary educational stratum, when compared with the results based on un-harmonized educational attainment (see upper panel). Among the tertiary educated, by contrast, the over-linkage of deaths remains. Linkage quality appears to be satisfactory only for the deaths of the majority population holding a secondary level diploma.

Emigration of foreigners around the retirement age does not importantly bias the results. The replicated assessment applied only to the population born in Switzerland and their linked deaths did not change the general age-patterns in *c(a)* (not shown). Nevertheless, the under-linkage of deaths to the primary school stratum of population aged less than 70 was less pronounced. Foreigners are indeed disproportionately concentrated in this age group. The remaining under- and over-linkage of deaths to the population aged 60 to 69 may be related to the SNC’s inadequate harmonization of the educational variables for people at early retirement ages. Given that deaths are less frequent at these ages, a small number of misclassified deaths leads to important rates of under or over-linkage – although the impact on the estimated life expectancy remains limited.

The misclassification of the population according to educational attainment (due to changes in the methodology of data collection) underestimated the educational gradient of life expectancy at age 60 in the 1990s. The differences between the tertiary and the primary educational strata were 3.3 years for men and 2.4 years for women when estimated with the linked deaths in the numerators and the original census-based at-risk populations in the denominators of the mortality rates. This gradient increased to respectively 4.0 and 2.8 years when using the harmonized educational data from the censuses and after correction of the number of linked deaths in the numerators (using the rates of over or under-linkage shown in the lower panel of Fig. 4).

### Andalusia

**Fig. 5:**
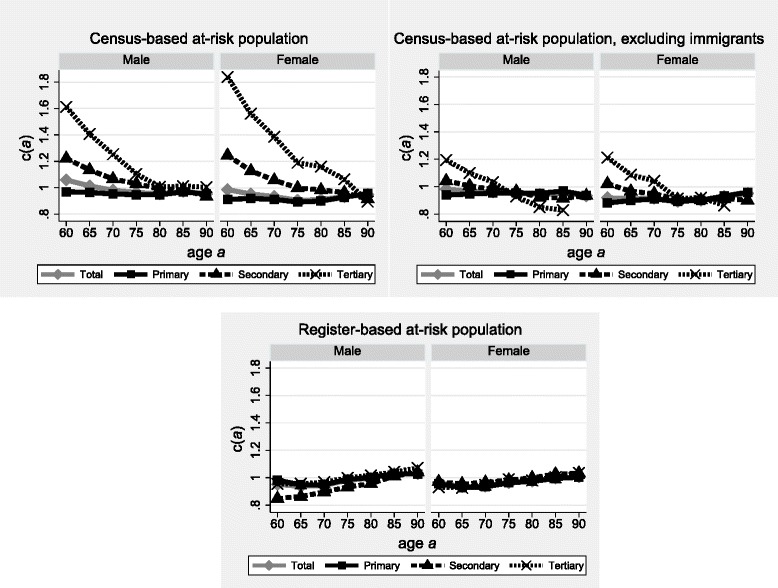
Linkage rates of deaths above age *a* according to sex and educational attainment, Andalucia (Spain), 2001–2011. Source: Base de Datos Longitudinal de Población Andalusia 2002–2011. Note: educational attainment as based on census and register data

The upper panel of Fig. 5 shows the results of the Andalusian assessment of linked deaths in the 2000s and population data from the 2001 and 2011 Censuses. The trends in *c(a)* for the total male and female populations decrease linearly over the ages 60 to 75 (from 99% to 90% among women, and from 107% to 96% among men). This indicates that the growth rates are over-estimated at these ages. Yet the results differ by educational attainment. For the lowest educational stratum, representing the large majority of the population, the sets of *c(a)* form a rather straight line slightly below unity for men and situated at around 90% for women. The over-estimation of growth rates in fact only concerns the (minority) tertiary and, to a lesser extent, the secondary educational stratum, and is more pronounced among women than among men.

The decreasing patterns of *c(a)* over age may be related to the important immigration peak around the retirement age in Andalusia. Migrant workers who returned from other regions of Spain or Europe tend to be more highly educated than the resident population. The natural and cultural amenities of Andalusia also attract many foreign retirees. The middle panel of Fig. 5 shows the results based on a new set of growth rates and population-based numbers of survivors, which have been re-estimated after exclusion of intercensal immigrants from the 2011 Census population. The declining trends in *c(a)* for the two higher educated strata is less pronounced but remains – especially for the tertiary educated population. This implies that problems with the educational variable or biases in the sampling-weights of the 2011 Census are more important.

The question about educational attainment changed slightly between the 2001 and the 2011 Censuses (with two new response items in 2011). Table 3 confirms that people who have been enumerated in both years have responded differently over time. The rate of congruence in the information about educational attainment between the two censuses is lowest for those who indicated a secondary diploma in 2001 (57% and 59% among women and men, respectively): more than a third declared a lower level in 2011, with the remainder having indicated a higher degree. Among the lowest and highest educated, the rates of “educational mobility” are at least 14%. Unfortunately, we are unable to estimate the impact of these problems on the linkage rates *c(a)* (because only the 2001 population was exhaustively enumerated).^4^

**Table 3:** Intercensal survivors’ stated educational attainment at the 2011 Census as a percentage of their self-classification at the 2001 Census, linked persons aged 60 and older by sex, Andalusia 2001 and 2011

Educational attainment in 2001	Educational attainment in 2011 (in %)						% distr. 2011	% distr. at the 2001 Census
	Primary or less	Secondary	Tertiary	Total	Numbers	Weighted numbers		
Men								
Primary or less	86	14	1	100	41,067	418,119	62	77
Secondary	34	59	7	100	14,193	172,539	26	17
Tertiary	3	14	83	100	6257	79,625	12	6
Total							100	100
Women								
Primary or less	89	10	0	100	58,105	626,658	74	86
Secondary	38	57	5	100	13,606	168,734	20	11
Tertiary	4	15	81	100	3752	48,813	6	2
Total							100	100

Source: linked Censuses 2001 and 2011

Table 3 also shows that the educational structure of the weighted 2011 Census sample population significantly differs from that of the exhaustive Andalusian population enumerated in 2001. The tertiary and secondary educated strata are over-represented in 2011 (respectively 12% and 26% among men, against 6% and 17% in 2001). This inadequate extrapolation of Andalusian statistics by the (national-level) sample weights leads to an over-estimation of the growth rates used to extrapolate the future cohort deaths of the secondary and tertiary educated strata from the linked period deaths. This would explain the decreasing trends in *c(a)* over age for these groups (see upper two panels of Fig. 5). The primary stratum, by contrast, is underrepresented in 2011, relative to 2001, which explains its increasing age-specific trend in *c(a)*.

The comparison of the results based on the at-risk population according to the censuses (upper panels of Fig. 5) with the results based on the annual population register (lower panel of Fig. 5) confirms that the 2011 Census misclassifies and misestimates the at-risk population (we are unable, however, to assess the respective importance of the two biases). The trends in linkage rates over age change radically. Education-specific estimates of *c(a)* based on register data are now only slightly below unity, similar to each other, and converge to unity at the highest ages. Thus, linkage precision is in fact satisfactory. The slightly increasing trends in *c(a)* over age point to a slight under-estimation of growth rates, which may be explained by the incomplete capturing of immigration flows by the population register. This seems to be particularly the case for men holding a secondary educational level.

In contrast to Switzerland, the educational mortality gradient in Andalusia was overestimated due to the misclassification and misestimation of the at-risk populations: the range of life expectancy at age 60 was at least four years based on census data in the denominator of mortality rates, but less than three years based on the annually updated register.

## Discussion

Socioeconomic gradients of mortality are increasingly documented based on probabilistically linked mortality and population data. However, their validity is often taken for granted. We assessed the plausibility of the numerators and denominators of old age mortality rates according to educational attainment from a demographic perspective in four different data contexts across Europe. Using the SEG method, we evaluated the consistency of the population data, corrected (where possible) the misclassification or misestimation of the at-risk populations, and estimated the rate of death linkage by educational attainment.

The main conclusion of this assessment is that the misclassification and misestimation of the at-risk population according to educational attainment were the main factors in biased mortality rates. The precision of death linkage according to educational attainment appeared to be lower in Switzerland and Andalucia, when compared to Finland and Lithuania, because the former two countries cumulate problems in the population data (misclassification and misestimation). Yet the replicated assessments using the harmonized information on educational attainment of the Swiss population, and the more precise estimates of their size in Andalucia, confirmed a high precision of the death linkage – even though a probabilistic procedure has been applied in Switzerland. Mortality estimates for the minority groups are most affected by problems in the population data, as even small numbers of misclassified individuals from other groups or incorrect weighting factors have a larger impact on the estimate of the at-risk population.

Based on this analysis, the SEG method can be recommended to assess the plausibility of socioeconomic mortality gradients derived from linked data. However, the linked database must fulfil a certain number of criteria for the method to provide useful information. Firstly, the underlying mathematical relationships between two successive counts of living individuals and of the deaths during the interval only hold when the linkage relies on all records of deaths and population (or on two equally representative samples). Secondly, when death registration and population enumeration are incomplete, results may be confounded by differential levels of completeness according to socioeconomic characteristics. In the high-quality statistical systems of OECD countries, however, the SEG method provides unambiguous information on the misclassification of linked deaths according to socioeconomic status (i.e., linkage precision), as well as on the misclassification or misestimation of the at-risk population over time. Thirdly, the ratios *c(a)* unambiguously estimate the linkage precision for different socioeconomic strata only when all deaths have been matched with population records (or when unlinked deaths have been imputed). In the presence of remaining unlinked deaths, the education-specific linkage precision may be assessed by comparison of the respective *c(a)* against the reference estimate computed for the total population (assuming that linkage sensitivity does not differ according to socioeconomic status).

Although death distribution methods have been extensively used in contexts of incomplete statistics, and have been validated on empirical and simulated data of known quality, a note of caution is necessary. The confidence intervals of the results increase strongly with the level of registration completeness or linkage precision [14, 15]. Moreover, the results provide an average estimate of the over- and under-linkage of deaths having occurred over the entire intercensal period, whereas the quality of death linkage usually decreases with distance from the date at which the population data have been collected. Nevertheless, death distribution methods provide valuable first indications of problems with the data in order to justify a more detailed investigation of their quality.

## Conclusion

The SEG method provides a powerful and low-cost tool to ensure an accurate monitoring of socioeconomic mortality gradients based on linked data and, thus, to enhance public health and develop social policies. As shown for the first time by this study, the quality of the denominator of the mortality rates matters and can be assessed. If the problems that bias the denominator and violate the simplifying assumptions of the SEG method can be solved (i.e., misclassification/misestimation of the at-risk population and the migration bias), the precision of death linkages can be estimated without knowing the true matching status among a subset of records. Furthermore, routine validations are greatly facilitated by the method’s limited requirements in terms of in-put data (i.e., age-specific counts of linked deaths and unlinked population disaggregated according to socioeconomic status). It can be recommended to use this approach a) to validate the results on changes in mortality differentials and b) to estimate a possible degree of uncertainty attributable to changes in classification of socioeconomic variables in different censuses.

The lessons from this study also have implications for the production and the use of linked mortality data. More attention should be directed towards the quality of the information on socioeconomic status, as well as of the estimation of at-risk populations for mortality rates. The Swiss results highlight the importance of identifying intercensal survivors through a linkage of individuals observed at the beginning and end of the period, before probabilistically matching the deaths to the remaining population at the first census. This not only increases the precision of the death linkage (due to fewer pairs of records with identical matching probabilities), but also enables one to harmonize information about the at-risk population recorded at different points in time. The comparability of time series of socioeconomic mortality gradients can thus be ensured.

Moreover, there is a trend in statistically developed countries to replace exhaustive census operations by sample censuses. In this context, the Andalucian linkage strategy can be recommended (i.e., first linking the deaths to the population register, followed by a linkage of the population register to the census in order to retrieve information on socioeconomic status), as it avoids the misestimation of the at-risk population according to socioeconomic status. Finally, the analyst may use the rates of education-specific over- and under-linkage as estimated by the SEG method to adjust the numbers of deaths and get accurate mortality estimates (if the precision of the linkage procedure cannot be improved further). Otherwise, it is advisable to regroup the most problematic (minority) groups in larger categories or to exclude them from the analysis.

### Methodological appendix

We use the Synthetic Extinct Generation (SEG) method, which has been designed to estimate the rate of completeness of death registration, relative to the completeness of census enumeration [20, 21]. Completeness of death registration is estimated by the ratio between the sum of all cohort deaths above exact age *a* – as approximated from the registered number of period deaths above *a* in a given period *t*, and the number of cohort members reaching age *a* in *t*.

**Fig. 6:**
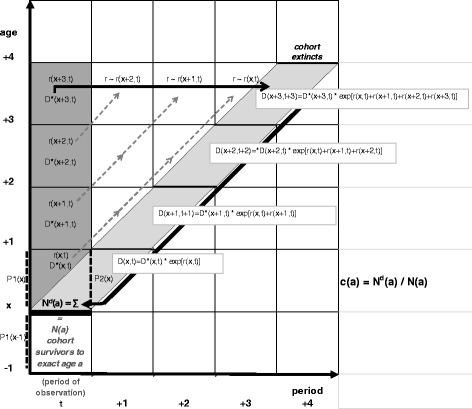
Schematic representation of the SEG method in a Lexis-diagram. Note: observed entities in period *t* are shown in grey, approximated future cohort deaths in blue. For the legend, see text

The number of cohort deaths above exact age *a* can be approximated by assuming a stable population: i.e., only the number of births is allowed to vary over time, with constant age-specific risks of mortality and no migration during the observation period *t*. The age-specific completeness of the censuses or population register is also assumed to be constant over time. As two adjacent births cohorts get older in this context (i.e., being subject to the same age-specific mortality), their numbers of survivors *and* of deceased will differ by the same rate at all ages. This rate will be equal to the growth rate in the cohort radixes, as constituted by the number of births in the past years. From a period perspective, therefore, the growth rate *r(x)* of the population at age *x* in *t* are proxies for the growth rates at the higher ages *x + y* in future years *t + y* (see Fig. 6). Consequently, the number of future deaths among the cohort who reached exact age *a* in *t* (*N* ^*d*^ *(a)*; light gray color in Fig. 5) can be approximated by multiplying, first, the number of deaths at age *x + y* (D*(x + y)) observed in *t* and, second, the exponentiated sum of the population growth rates *r(x)* at the ages *x* to *x + y* in *t* (both dark grey color in Fig. 6). In other words, the number of future cohort deaths is approximated by adjusting the number of current period deaths for the demographic history of the population [14, 23]:

The fraction of deaths above age *a* in *t* that is over- or under-registered by vital statistics (*c(a)*) is given by the ratio of the approximated numbers of all future deaths among the cohort reaching age *a* in *t* (*N* ^*d*^ *(a)*) divided by a population-based estimate of the same cohort survivors in *t* (*N(a)*). This number can be approximated from two successive (census or register) age-distributions (*P1(x-1)* and *P2(x)* in Fig. 6).

Linearly increasing or decreasing trends in *c(a)* over successive ages indicate that the age-specific growth rates are under- or over-estimated, respectively. The intercensal growth rates *r(x)* will be under- or over-estimated when the population at the second census is under- respectively over-enumerated, relative to the first one. Under- or over-estimation of growth rates may also result from the depletion respectively swelling of the population due to intercensal migration. As this negative or positive bias in *r(x)* is cumulated downwards by age to estimate *N* ^*d*^ *(a)*, *c(a)* is more strongly under- or over-estimated at younger ages, when compared to older ages. This yields to a linearly increasing respectively decreasing trend in *c(a)* with age.

We used data grouped by 5-year age intervals in order to increase robustness of the results, and applied the United Nations’ variant of the method designed for 10-year census intervals [23]. (The UNFPA variant allows applications over any other interval - at the expense of additional approximations [24]). This meant that we had to extrapolate the sex-, age-, and education-specific numbers of deaths for one month in Lithuania and for one year in Andalucia to match the 10-year intercensal periods. As deaths are highly concentrated in older ages, we defined the last open-ended age group as being either 90 or 95 years and above – depending on the numbers of deaths by country.

The initial estimation of *N* ^*d*^ *(a)* for the last open-ended age group relies on a special formula [20] and an external estimate of remaining life expectancy as input. The same country-specific official estimates of life expectancy for the total male and female populations were used for all educational strata.

## Abbreviations

*c(a)*: Linkage rate of deaths above age *a*
ID: Identification number
*N(a)*: Population-based estimate of cohort survivors to age *a* in a given period
*N*^*d*^*(a)*: Deaths-based estimate of cohort survivors to age *a* in a given period
OECD: Organisation for Economic Co-operation and Development
SEG: Synthetic extinct generation
SFSO: Swiss Federal Statistical Office
SNC: Swiss National Cohort

## Footnotes

1 Inconsistencies in the information on education were possible for some cases, e.g. higher educational attainment in 2000 compared to 1990, especially for certain age groups, but not the other way round.

2 In Switzerland, a significant share of missing values have been imputed by the Statistical Office (especially at the 2000 Census), and we allocated the remaining numbers among each sex and age-specific subpopulation proportionally. In Finland and Lithuania, missings were regrouped with the lowest educated stratum based on previous tests of similarity. In Andalucia, all missings were imputed by the statistical office.

3 To test the effect of emigration of native Swiss, one may adjust the sex-, age-, and education-specific population counts in 2000 for the numbers of Swiss nationals who moved abroad since 1990. However, we are unable to perform this sensitivity analysis as only total numbers are published by the Swiss Federal Statistical Office; the small number of 76,000 is unlikely to introduce significant biases.

4 We experimented with different redistributions across our three broad education categories of the counts of the two new detailed educational classes in the 2011 Census, but results changed only marginally (not shown).
