# Erratum to: on the plausibility of socioeconomic mortality estimates derived from linked data: a demographic approach

**DOI:** 10.1186/s12963-017-0148-y

**Published:** 2017-08-17

**Authors:** Mathias Lerch, Adrian Spoerri, Domantas Jasilionis, Francisco Viciana Fernandèz

**Affiliations:** 1 Max Planck Institute for Demographic Research, Demographic Research Centre, Vytautas Magnus University, Rostock, Germany; 2 Institute of Social and Preventive Medicine, University of Bern, Bern, Switzerland; 3 Institute of Statistics and Cartography of Andalusia, Andalusia, Spain

## Erratum

The original article [1] was corrected. An incorrect version of Figure 3 was initially published. The correct version of Fig. 3 is also shown below.

**Fig. 3:**
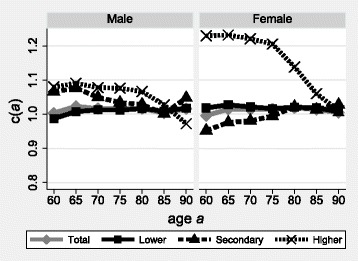
Linkage rates of deaths above age *a* according to sex and educational attainment, Lithuania, 2001–2011. Source: Census-linked mortality data Lithuania, 2001–2011. Note: educational attainment as based on censuses
